# Estimation of significance thresholds for genomewide association scans

**DOI:** 10.1002/gepi.20297

**Published:** 2008-04

**Authors:** Frank Dudbridge, Arief Gusnanto

**Affiliations:** MRC Biostatistics Unit, Institute for Public HealthCambridge, United Kingdom

**Keywords:** multiple testing, permutation test, Bonferroni, eigenvalue

## Abstract

The question of what significance threshold is appropriate for genomewide association studies is somewhat unresolved. Previous theoretical suggestions have yet to be validated in practice, whereas permutation testing does not resolve a discrepancy between the genomewide multiplicity of the experiment and the subset of markers actually tested. We used genotypes from the Wellcome Trust Case-Control Consortium to estimate a genomewide significance threshold for the UK Caucasian population. We subsampled the genotypes at increasing densities, using permutation to estimate the nominal *P*-value for 5% family-wise error. By extrapolating to infinite density, we estimated the genomewide significance threshold to be about 7.2 × 10^−8^. To reduce the computation time, we considered Patterson's eigenvalue estimator of the effective number of tests, but found it to be an order of magnitude too low for multiplicity correction. However, by fitting a Beta distribution to the minimum *P*-value from permutation replicates, we showed that the effective number is a useful heuristic and suggest that its estimation in this context is an open problem. We conclude that permutation is still needed to obtain genomewide significance thresholds, but with subsampling, extrapolation and estimation of an effective number of tests, the threshold can be standardized for all studies of the same population.

## INTRODUCTION

The question of what strength of evidence should be considered significant has yet to be fully resolved in genetic association analysis. On the one hand, multiple testing issues arise in most studies, whether based on candidate genes or genomewide scans, with attendant issues of how to quantify the multiplicity, what error rate to control and which method to use [Manly et al., 2004]. On the other hand, even under strong control of the type–1 error, many associations have not been replicated and are thought to have been false positives. This disappointing aspect can be attributed to the use of traditional thresholds of significance, even after adjustment for multiple testing, which reflect over–optimistic prior belief in the tested hypotheses [Ioannidis, 2005]. In the current period of genomewide association scans, using a dense but incomplete panel of single nucleotide polymorphism (SNP) markers [Barrett and Cardon, 2006], such considerations take on greater importance owing to the high profile and cost of these studies.

Here we discuss some aspects of assessing significance in genomewide scans, and estimate a genomewide significance threshold for the UK Caucasian population using data from the recently completed Wellcome Trust Case-Control Consortium [2007] (WTCCC) study. We allow for a potentially saturated dense marker panel to distinguish the genomewide multiplicity of the experiment from the set of markers actually tested. This approach has a long history in linkage analysis [Morton, 1955; Lander and Kruglyak, 1995] but has generally not been taken up in association mapping. Our approach brings together previous ideas based on the hypothesized extent of multiplicity in the genome, with emerging marker data that allow this multiplicity to be estimated.

The multiple testing problem arises because, if many hypotheses are tested simultaneously, some test statistics will be surprisingly extreme, even if no associations exist. Multiple test procedures are designed to exercise control over the entire set of hypotheses, to prevent study–wide conclusions being drawn that could be attributed to chance alone. The family–wise error rate (FWER) is the probability of committing at least one type–1 error, and may be controlled in the weak sense, when all null hypotheses are true, or in the strong sense, when any subset of hypotheses is true [Hochberg and Tamhane, 1987]. More recently, the false discovery rate (FDR) [Benjamini and Hochberg, 1995] and variations [Efron and Tibshirani, 2002] have gained support, as we may tolerate some type–1 errors so long as they are a small proportion of the rejected hypotheses. Bayes factors have also been advocated to quantify the strength of evidence in each test [WTCCC, 2007]. Here we are not concerned with discriminating between different error measures, but note that when the number of false hypotheses is small, control of the standard FDR is close to weak control of the FWER [Benjamini and Hochberg, 1995]. Moreover, in this situation strong control of FWER is close to the best methods for weak control [Dudbridge and Koeleman, 2003]. Also when the evidence is strong and the power is high, there is a strong correlation between the Bayes factor and the *P*–value [Thomas and Clayton, 2004]. In what follows we will therefore consider strong control of FWER by the Bonferroni method, or its permutational equivalent [Westfall and Young, 1993], as a conservative baseline to which other methods can be calibrated.

The first well–known suggestion for a genomewide significance threshold for association was by Risch and Merikangas [1996]. They assumed a gene-centric study of 100,000 genes, with an average of five SNPs tested in each gene, leading to a Bonferroni significance threshold of 5 × 10^−8^ for one–sided tests of each allele. Although these assumptions are now obsolete, this threshold has proved remarkably durable, and valid in the sense that, to our knowledge, no association reported as exceeding it has proved a false positive. Another estimate for a gene-centric study assumed 30,000 genes, with 10 genes having normally distributed effects, leading to a threshold of 1 × 10^−6^ for a genewide test [Clayton, 2003; Thomas and Clayton, 2004].

More recently, attention has shifted from gene-centric studies to genomewide scans of anonymous SNPs. In this context the International HapMap Consortium [2005] used permutation tests of high density genotypes in 10 genomic regions to estimate an “effective number of independent tests” of 150 per 500 kbp. Scaling up to a 3 Gb genome suggests a significance threshold of 5.5 × 10^−8^ for two–sided tests of SNPs. The only threshold that has to date been rigorously applied to more than one scan is that of the WTCCC [2007]. Assuming 10^6^ independent regions of the genome, 10 disease–causing genes and average power 50% to detect an associated gene, posterior odds of 10:1 in favour of association would be achieved by a nominal *P*–value of 5 × 10^−7^.

With genomewide SNP data now available, permutation tests have been suggested to obtain appropriately adjusted *P*–values [Churchill and Doerge, 1994]. The difficulty is that standard permutation tests only account for the markers actually tested, whereas the multiplicity spans the whole genome. This is important because although *P*–values are the usual output of classical tests, the real quantities of interest are the posterior odds for association or the closely related false–positive reporting probability [Wacholder et al., 2004] and positive predictive value [Ioannidis, 2005]. For a class of tests significant when a statistic *T*>*t*, the posterior odds may be expressed in terms of the prior odds as

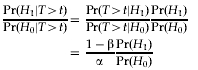
(1) where α, Beta are the type–1 and type–2 error rates of the test, respectively. Consideration of the prior odds is intrinsic to the Bayesian perspective, but it is also implicit in frequentist testing, if it is to be used responsibly.

Suppose one chose a single SNP at random, tested for association to a trait and obtained a *P*–value of exactly 0.05. As one test was performed, no correction for multiplicity is required, and from the frequentist perspective one may reject the hypothesis that no SNP is associated and invoke the closure principle to claim that the tested SNP is associated [Hochberg and Tamhane, 1987]. However, this is clearly absurd, as there was almost no prior reason to expect an association. In fact at standard error rates of α = 0.05 and Beta = 0.2, one would require prior odds of 2:1 against to obtain posterior odds of 8:1 in favor of association, or prior odds of evens to obtain posterior odds of 16:1. Usage of standard frequentist thresholds is only justified when the prior odds are in this order of magnitude, say between evens and 10:1 against.

Of course, tested SNPs are not chosen randomly, but the relative lack of success of candidate–gene association studies, apparently well powered for nominal significance, suggests that the prior odds have generally been exaggerated [Ioannidis, 2005]. However, a genomewide scan, for which SNPs are provided by a commercial supplier of genotyping chips, is in a sense little different from a random selection, except that the prior odds are substantially shortened. Yet, unless the chip has complete coverage of the genome, there remains a discrepancy between the number of SNPs tested and the actual prior odds of any SNP being associated. Our view is that a responsible use of *P*–value thresholds must reflect the multiplicity of the whole genome, rather than of the set of SNPs tested. This is in line with the previous approaches [International HapMap Consortium, 2005; WTCCC, 2007] and facilitates the comparison of results between different marker panels as well as those obtained by imputation methods that estimate the genotypes of markers not directly typed [Marchini et al., 2007]. However, permutation methods for multiple test correction only take account of the genotyped markers, and use of these methods with standard thresholds may, potentially, lead to over–optimistic interpretation of significant results.

Given these difficulties the Bayesian perspective seems attractive, but now one is faced with quantifying the prior odds. In fact, there is now sufficient background knowledge to allow this to be done rather objectively [Wachholder et al., 2004; Wakefield, 2007], and it can be argued that the major obstacle to widespread use of this paradigm is convention. Nevertheless, these approaches can only be validated through experience, and as a rule of thumb, naïve Bayesian analysis should result in similar conclusions to a frequentist analysis, if both are well calibrated.

With these arguments in mind, we therefore consider estimation of a frequentist *P*–value threshold that accounts for the multiplicity of the whole genome and can be used as a baseline for calibrating other approaches. We consider two approaches to estimating this threshold, accounting for the correlation within the genome: one based on a permutation scheme, and the other using linear algebra to estimate an effective number of tests directly from genotype data [Patterson et al., 2006]. We apply both methods to genotypes released by the WTCCC, extrapolating the results to complete saturation to obtain a genomewide threshold. We conclude by discussing some implications of these results in the light of the WTCCC and other studies.

## METHODS

### DATA

We used genotypes from the two control cohorts in the WTCCC [2007] study to sample the UK Caucasian population without disease association. Data were available for 1,485 blood donors (the National Blood Service (NBS) sample) and 1,504 members of the 1958 British Birth Cohort. We considered the two samples, both separately and combined. We analyzed the autosomal data only, for which genotypes were available for 490,032 SNPs on the commercial release of the GeneChip 500K Affymetrix array. We excluded genotypes whose posterior probability according to the CHIAMO algorithm [WTCCC, 2007] was less than 0.95: this excluded 2.3% of genotypes in the NBS and 1% in the 58BC samples. In order to ensure that all test statistics had the χ^2^ distribution under the null hypothesis, we only used SNPs for which there were at least 10 subjects having each genotype, leaving 334,773 SNPs in the NBS, 335,331 in the 58BC and 359,491 in the combined samples.

### PERMUTATION TEST

We estimated a genomewide significance threshold using a permutation procedure. We randomly designated half the sample “cases” and the other half “controls” and calculated the Armitage test of trend for differences in genotype frequency. *P*–values for all SNPs were sorted and the 1,000 smallest were recorded. This procedure was repeated 10,000 times.

The 5% quantile point of the minimum *P*–value represents the genomewide significance threshold at this marker density. To extrapolate to complete saturation, we randomly subsampled the SNPs over a range of lower densities by equivalently subsampling the sorted *P*–values independently for each permutation replicate. We used a uniform grid of 100 marker densities, and at each density the 5% point of the minimum *P*–value was recorded. We repeated the subsampling 100 times at each density and used the mean 5% point in subsequent analysis. At low densities, the SNPs are expected to be independent; hence, according to the Bonferroni law the 5% point is inversely proportional to the number of SNPs. At high densities we expect the 5% point to converge to an asymptote, reflecting the significance threshold for the whole genome.

Denote the proportion of SNPs subsampled by *x*_*i*_;*i* = 1,2, …, where *x*_*i*_<*x*_*i*+1_ and *x*_*i*_ are relative to the total number of SNPs used in the estimation. Denote the corresponding 5% quantile points *y*_*i*_. It follows that the effective number of independent tests defined by

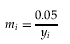
(2)
should be proportional to *x*_*i*_ when *x*_*i*_ is small and should converge to an asymptote as *x*_*i*_ grows large. To obtain these properties, we fit the Monod function to (*x*_*i*_,*m*_*i*_):

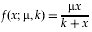

This model is not claimed to be exact, but it has been found to fit data well in applications such as modeling population growth with limited resources [e.g. Cohen and Gürtler, 2001]. The parameters of this model are μ, the limit as *x* → ∞, and *k*, the half–saturation parameter representing the value of *x* for which *f*(*x*) = μ/2. We estimated the parameters by least squares to give the genomewide significance threshold as

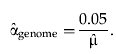
(4)
We used a non–parametric bootstrap to estimate confidence intervals for 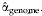;_*genome*_. We resampled the minimum *P*–values with replacement from the permutation replicates, and for each resampling we subsampled SNPs, fitted the Monod function and calculated 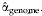. We estimated 95% confidence intervals for 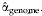; *k* and 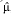 from 1,000 bootstrap samples.

### EFFECTIVE NUMBER OF TESTS

Permutation procedures are time consuming, and an attractive alternative is to estimate an effective number of independent tests directly from the genotype correlation matrix. A moment–based estimator was recently proposed by Patterson et al. [2006] based on the eigenvalues of the correlation matrix. This estimator has good properties in the context of detecting population structure, but we wished to see whether it is equally useful for correcting multiplicity. Let

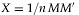
(5)
where *n* is the number of markers and *M* is a normalized matrix of genotypes with one row per subject and one column per marker [for details, see Patterson et al., 2006]. Denoting the eigenvalues of X by λ_1_,…,λ_*m*_, where *m* is the number of subjects, the effective number of tests is estimated by

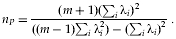
(6)
As for the permutation test, we estimated *n*_*P*_ for a grid of subsampling densities. We calculated *n*_*P*_ for each chromosome and summed to obtain a genomewide estimate.

### BETA DISTRIBUTION

If there really is an effective number of independent tests *n*_*E*_, then the minimum *P*–value should follow a Beta distribution with parameters (1,*n*_*E*_), as [Šidák, 1967]

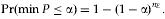
(7)
We fitted the Beta distribution to the minimum *P*–value of the permutation replicates, with the first parameter set to 1 and also with both parameters free. This would allow us to test whether the minimum *P*–value is consistent with an effective number of independent tests, by testing whether the first parameter is 1 [Dudbridge and Koeleman, 2004], and whether Patterson's estimator is accurate, by testing whether *n*_*p*_ = *n*_*E*_. The moment estimators for the parameters of the Beta (*a*,*b*) distribution are

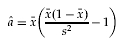
(8)

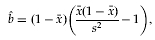
(9)
where 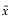 and *s*^2^ are the sample mean and variance of observations, respectively. When *a* is set to 1, the moment estimate of *b* is

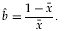
(10)
We used the moment estimates as starting points for numerical maximum likelihood estimation, using the optim function in R.

## RESULTS

**Fig. 1:**
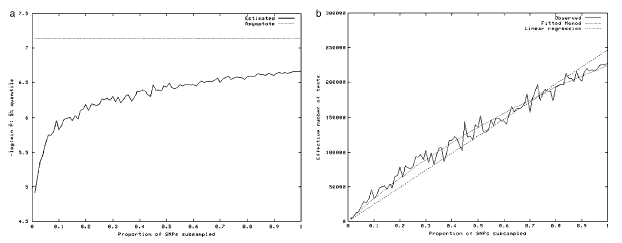
(a) Significance threshold as a function of marker density in combined NBS and 58BC sample from permutation procedure. At current density (359K single nucleotide polymorphisms typed) the significance threshold is about 2.2 × 10^−7^. The dotted line shows the estimated asymptote of 7.2 × 10^−8^. (b) Fitted Monod function to the effective number of tests associated with the significance threshold. At infinite density the number of tests is estimated at 693,138 giving the asymptote in (a).

For the permutation procedure, Table I gives the estimated asymptote 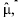, half–saturation parameter 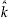 and genomewide significance threshold 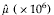 for the NBS and 58BC samples separately and combined. It is clear that the estimates are similar for the separate cohorts, so they may be combined to give greater precision. Figure 1(a) shows the threshold for 5% family–wise error plotted as a function of marker density for the combined samples. Figure 1(b) shows the corresponding effective numbers of tests together with the fitted Monod function. The curve is a good fit, but it is clear that it is not at its asymptote at the current density, although some curvature is apparent when compared with the linear regression line. The estimated asymptote was 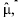 = 651,550, which increases to 693,138 assuming that the autosomes comprise 94% of the total genome length. This gives our estimated genomewide significance threshold as

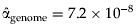
(11)
with 95% bootstrap confidence interval (6.3–8.9) × 10^−8^.

**TABLE I:** Fitted asymptotes 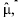 and half–saturation parameters 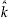 of Monod functions

	NBS	58BC	NBS+58BC
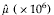	5.29(4.25–6.07)	4.96"(4.14–5.70)	6.52(5.26–7.43)
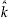	1.73(1.35–1.92)	1.58(1.28–1.82)	1.88(1.47–2.11)
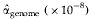	8.9(7.7–11.3)	9.5(8.3–11.4)	7.2(6.3–8.9)

Estimated genomewide significance thresholds 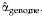 are calculated as 0.047/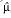, assuming that the autosomes comprise 94% of the total genome length.

NBS: National Blood Service.

58BC: 1958 British Birth Cohort.

The half–saturation parameter was estimated as 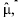 = 1.88 (1.47–2.11), indicating that at about twice the current number of markers, only half the total multiplicity will be accounted for. This does not mean that current marker panels have insufficient coverage, rather that permutation correction based only on typed markers may be too optimistic; we return to this point in the Discussion.

**Fig. 2:**
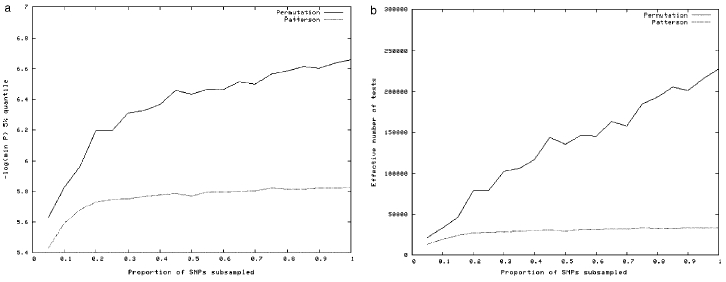
(a) Significance thresholds from permutation procedure and Patterson's estimate of the effective number of tests. At current marker density, the estimates differ by an order of magnitude. (b) The effective numbers of tests based on the permutation procedure and Patterson's estimator. At current marker density, Patterson's estimate is too low (33,279) compared to that of the permutation procedure (227,838).

For Patterson's estimator, Figure 2 shows the 5% family–wise error threshold and effective number of tests compared to the permutation procedure, over a uniform grid of 20 marker densities. There is clearly a wide discrepancy and at the current marker density the estimate is an order of magnitude too low: 33,279 compared to 227,838 for the permutation scheme, even though the latter allowed for correlation between chromosomes. The use of *P*–value thresholds based on this estimator will therefore inflate the false–positive rate. This result is not entirely surprising, as we have previously noted that the effective number of tests, if it exists, is a function of both the significance threshold and also of the type of analysis [Dudbridge and Koeleman, 2004]. Thus, it is not unexpected that an estimator that works well for analysis of population structure is not accurate for Bonferroni corrections.

**Fig. 3:**
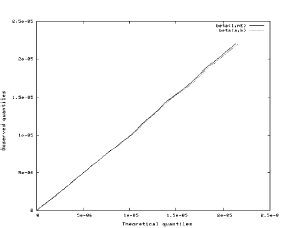
Quantile–xsquantile plot comparing fitted Beta distributions with minimum *P*-values from permutation replicates.

However, the fitted Beta distributions do suggest that an effective number of tests exists and could be useful. Figure 3 compares the empirical distribution of the minimum *P*–value for the combined samples, to the fitted Beta (1,*n*_*E*_) and Beta (*a*,*b*) distributions. Both Beta distributions are clearly a good fit to the observed data. The maximum likelihood estimate 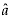 = 0.97 is close to 1; the null hypothesis of equality was formally rejected (*P* = 0.01), but this is not surprising given our high power to reject strict equality, and the test was not significant in the separate NBS and 58BC samples. This is in line with our results on an early version of HapMap [Dudbridge and Koeleman, 2004], in which the test of equality was extremely significant, suggesting that the effective number of tests is a worse fit at higher marker densities. The effective numbers of tests were similar to those estimated from the permutation procedure for both cohorts (Table II).

**TABLE II:** Estimated effective numbers of tests corresponding to the 5% point of the minimum P–value, from fitting Beta (1,*n*_E_) distribution and from permutation scheme

	NBS	58NC	NBS+58BC
Beta Distribution	195,469	199,334	216,457
Permutions	195,539	193,505	227,838

## DISCUSSION

To set a baseline for multiplicity adjustments, we have estimated a genomewide significance threshold of about 7.2 × 10^−8^ for two–sided tests of single SNPs in the UK Caucasian population. This is similar to the threshold suggested by the International HapMap Consortium [2005], which is reassuring as that study took a complementary approach. They extrapolated near-saturated local regions to the whole genome, whereas we took sparse data over the whole genome and extrapolated to complete saturation. Nevertheless, our estimate is not precise: in contrast to previous approaches we are able to report a confidence interval, which reflects the variation due to the finite number of permutations and random subsampling of SNPs. The half–saturation parameter is about twice the current number of markers, suggesting that the current marker density reflects less than half the total multiplicity in the genome. This does not imply that the marker panels have insufficient coverage, for it has been estimated that up to 80% of the variation in the genome is correlated to current panels [Barrett and Cardon, 2006]. Rather, the statistical cost of obtaining all the remaining information is high in relation to what is already available, and the number of markers that give near-complete saturation could be much lower. Nevertheless, it is clear that denser chips are needed to obtain more accurate estimates of significance thresholds, and it is meanwhile prudent to treat these thresholds as no more than informal guidelines.

We have relied on permutation testing to estimate significance thresholds, as this preserves the correlation structure in the sample. Similar results from the NBS and 58BC samples suggest that sampling variation in the correlation structure is negligible. Permutation tests are time consuming, and a convenient alternative is to estimate an effective number of tests from the genotype correlation matrix. Our results from fitting Beta distributions indicate that such an effective number can be found, at least at the current density, but we found that Patterson's moment estimator was an order of magnitude too low for correcting the minimum *P*–value. It is an open problem to directly estimate the appropriate correction for minimum *P* from genotype data.

The WTCCC study is widely held to have been a success, yet the *P*–value threshold used by that study is an order of magnitude higher than our estimate. Why were there not many false positives? One reason is that the rationale for their choice of 5 × 10^−7^ was based on prior odds for a region being associated, rather than a single SNP, and adjustment towards single SNP tests could bring the estimate closer to ours. Another practical aspect is that, for reasons of quality control, the study only followed up regions with multiple SNPs showing evidence of association. A formal combination of evidence from neighboring SNPs could produce stronger *P*–values exceeding those of the single SNPs. Finally, of the 21 single-SNP associations reported by the study, only three had *P*–values less than 5 × 10^−7^ but greater than 8 × 10^−8^, so that chance may have played a role.

Nevertheless, at the time of writing the WTCCC reported successful replication of 10 associations from 11 attempts. Assuming that the failed replication was a false positive, this does not constitute success by classical measures: a family–wise type–1 error occurred, and the false discovery proportion was nearly 10%. Only the Bayesian interpretation that posterior odds for association are 10:1 seems satisfactory. Therefore, the fact that the study is considered a success throws light on how multiplicity is interpreted in practice. Family–wise error is too conservative for rejecting individual hypotheses, and the FDR can be higher than traditional type–1 error rates. Posterior odds of 10:1 seem acceptable, which justifies the use of *P*<0.05 for hypothesis testing provided that the prior odds are close to evens. Such a prior is reasonable for genomewide association scans as long as the whole genome is considered rather than limited subsets of markers.

If family–wise error is too conservative, then do we need a genomewide significance threshold? We have argued that for the global null that no locus is associated, strong control of FWER is an acceptable proxy for weak control, FDR or Bayes factor approaches. Thus, if no locus is genomewide significant, we may conclude that no associations have been found, whereas if some loci are genomewide significant, other approaches can be used to decide which should be declared associated. It may be argued that the global null is not of interest, as the entire study is predicated on it being false. However, we feel that a significant global test is evidence that there is sufficient signal in the data to distinguish true from false positives. Given the uncertainty over what thresholds should be applied, the common pragmatic approach is to follow up associations in rank order, and this can be justified by a significant global test. Estimation of a genomewide significance threshold gives a baseline that can justify both pragmatic and principled approaches to selecting loci for follow–up.

We have only considered common SNPs represented on the GeneChip 500k array, and moreover only those for which the χ^2^–distribution is accurate. Mathematically this is not a serious problem: provided uniformly distributed *P*–values can be obtained, our approach applies to any variants. However, it is possible that rare variants follow a different linkage disequilibrium (LD) structure, because they are likely to be younger or under different selection pressures than common variants. Our results strictly apply only to common variation, and the implicit assumption that common variation occurs at arbitrarily fine scales may be unreasonably conservative. The similarity of our estimate to that of the International HapMap Consortium [2005] suggests that this assumption has not had a major impact on our results.

We have also made no assumption about which SNPs are more likely to be associated. One commonly held view is that non–synonymous or other coding SNPs are more likely to be associated than random SNPs, but this view is by no means universal, and the evidence is only now accruing through the first generation of scans. Furthermore, in a linkage disequilibrium scan of tag SNPs, it is harder to distinguish functional from non–functional SNPs. Another view is that SNPs in longer blocks of LD are more likely to be associated [Pe'er et al., 2006]. However, in a scan of randomly chosen SNPs, such as those considered here, this prior is likely to be attenuated by the fact that longer blocks of LD are likely to contain more genotyped SNPs [I. Pe'er, Personal Communication]

Strictly speaking, our results apply only to the UK Caucasian population, but we should expect similar results in other outbred populations of the same age. The HapMap data could be used to apply our methods to African and Asian populations, using a denser map of SNPs, but because of the small sample size we are not confident that the genomewide distribution of statistics from permutation samples is the same as would be applicable to a large sample. We therefore restricted our study to a large sample that has been used for a real genomewide association scan.

We have shown that previous proposals for genomewide significance have been in the right order of magnitude. It seems clear that, in a Western population, any *P*–value less than say 5 × 10^−8^ can be regarded as convincingly significant. We rely on permutation testing to estimate significance thresholds, but these should be adjusted to reflect the genomewide multiplicity. Estimation of an effective number of tests remains an open problem but one which has potential to considerably reduce the computational burden. The next generation of genotyping chips should allow more accurate estimation of significance thresholds with application to a wider range of genomic variation.

## References

[b1] Barrett JC, Cardon LR (2006). Evaluating coverage of genome-wide association studies. Nat Genet.

[b2] Benjamini Y, Hochberg Y (1995). Controlling the false discovery rate—a practical and powerful approach to multiple testing. J R Stat Soc B.

[b3] Churchill GA, Doerge RW (1994). Empirical threshold values for quantitative trait mapping. Genetics.

[b4] Clayton DG (2003). *P*-values, false discovery rates, and Bayes factors: how should we assess the “significance” of genetic associations?. Ann Hum Genet.

[b5] Cohen JE, Gürtler RE (2001). Modeling household transmission of American trypanosomiasis. Science.

[b6] Dudbridge F, Koeleman BP (2003). Rank truncated product of *P*-values, with application to genomewide association scans. Genet Epidemiol.

[b7] Dudbridge F, Koeleman BP (2004). Efficient computation of significance levels for multiple associations in large studies of correlated data, including genomewide association studies. Am J Hum Genet.

[b8] Efron B, Tibshirani R (2002). Empirical Bayes methods and false discovery rates for microarrays. Genet Epidemiol.

[b9] Hochberg Y, Tamhane AC (1987). Multiple Comparison Procedures.

[b10] International HapMap Consortium (2005). A haplotype map of the human genome. Nature.

[b11] Ioannidis JP (2005). Why most published research findings are false. PLoS Med.

[b12] Lander ES, Kruglyak L (1995). Genetic dissection of complex traits: guidelines for interpreting and reporting linkage results. Nat Genet.

[b13] Manly KF, Nettleton D, Hwang JT (2004). Genomics, prior probability, and statistical tests of multiple hypotheses. Genome Res.

[b14] Marchini J, Howie B, Myers S, McVean G, Donnelly P (2007). A new multipoint method for genome-wide association studies by imputation of genotypes. Nat Genet.

[b15] Morton NE (1955). Sequential tests for the detection of linkage. Am J Hum Genet.

[b16] Patterson N, Price AL, Reich D (2006). Population structure and eigenanalysis. PLoS Genet.

[b17] Pe'er I, de Bakker PI, Maller J, Yelensky R, Altshuler D, Daly MJ (2006). Evaluating and improving power in whole-genome association studies using fixed marker sets. Nat Genet.

[b18] Risch N, Merikangas K (1996). The future of genetic studies of complex human diseases. Science.

[b19] Šidák Z (1967). Rectangular confidence regions for the means of multivariate normal distributions. J Am Stat Assoc.

[b20] Thomas DC, Clayton DG (2004). Betting odds and genetic associations. J Nat Cancer Inst.

[b21] Wacholder S, Chanock S, Garcia-Closas M, El Ghormli L, Rothman N (2004). Assessing the probability that a positive report is false: an approach for molecular epidemiology studies. J Nat Cancer Inst.

[b22] Wakefield J (2007). A Bayesian measure of the probability of false discovery in genetic epidemiology studies. Am J Hum Genet.

[b23] Wellcome Trust Case Control Consortium (2007). Genome-wide association study of 14,000 cases of seven common diseases and 3,000 shared controls. Nature.

[b24] Westfall PH, Young SS (1993). Resampling-Based Multiple Testing: Examples and Methods for *P*-value Adjustment.

